# Noncytotoxic functions of killer cell granzymes in viral infections

**DOI:** 10.1371/journal.ppat.1009818

**Published:** 2021-09-16

**Authors:** Lisanne C. de Jong, Sandra Crnko, Toine ten Broeke, Niels Bovenschen

**Affiliations:** 1 Radboud University, Nijmegen, the Netherlands; 2 Department of Pathology, University Medical Center Utrecht, Utrecht, the Netherlands; 3 Center for Translational Immunology, University Medical Center Utrecht, Utrecht, the Netherlands; Yale University School of Medicine, UNITED STATES

## Abstract

Cytotoxic lymphocytes produce granules armed with a set of 5 serine proteases (granzymes (Gzms)), which, together with the pore-forming protein (perforin), serve as a major defense against viral infections in humans. This granule-exocytosis pathway subsumes a well-established mechanism in which target cell death is induced upon perforin-mediated entry of Gzms and subsequent activation of various (apoptosis) pathways. In the past decade, however, a growing body of evidence demonstrated that Gzms also inhibit viral replication and potential reactivation in cell death–independent manners. For example, Gzms can induce proteolysis of viral or host cell proteins necessary for the viral entry, release, or intracellular trafficking, as well as augment pro-inflammatory antiviral cytokine response. In this review, we summarize current evidence for the noncytotoxic mechanisms and roles by which killer cells can use Gzms to combat viral infections, and we discuss the potential thereof for the development of novel therapies.

## Introduction

Cytotoxic lymphocytes (CLs), including natural killer (NK) cells, NKT cells, γδ TCR cells, and cytotoxic T lymphocytes (CTLs), represent the major defense force against viral infections. They secrete pro-inflammatory cytokines, particularly interferons (IFNs), to induce a systemic antiviral state, and they can mediate target cell death by producing serine proteases (granzymes (Gzms)) and pore-forming proteins, including perforin. Gzms and perforin are expressed in granules inside all CL subtypes and secreted by fusion of these granules with the cell membrane. CL subsets express different levels of each Gzm, which are prone to change upon CL differentiation and activation, pointing to their distinct roles in immune responses [1,2]. Upon cognate binding and CL activation, Gzms are released into the immunological synapse between CL and the target cell. Perforin pores help Gzms to enter the target cell [2,3], after which they can cleave proteins, e.g., involved in apoptosis, cytokine response, and/or the viral or host cell life cycle. The mechanism underlying perforin-mediated Gzm entry is debatable. Gzms can enter host cells through perforin pores in the plasma membrane and/or Gzms, and perforin can enter target cells via (receptor-mediated) endo/pinocytosis after which perforin plays a yet to be established role in liberating Gzms from endosomes [3,4]. Gzms can also exist outside cells in the extracellular microenvironment likely through leakage from the immunological synapse [5]. Alternatively, these extracellular Gzms can also derive from expression and secretion by CLs or other cells, including monocytes, macrophages, dendritic cells (DCs), mast cells, or basophils [6]. Extracellular Gzms have been demonstrated to play roles in matrix remodeling, wound healing, and augmenting inflammation and cytokine response [6]. Mechanistically, the latter can occur through cleavage of membrane receptors [7] or via cleavage of substrates inside target cells [6,8]. How these extracellular Gzms enter the target cell cytoplasm largely remains unknown, although cell surface heparin sulfate proteoglycans and endocytosis receptors have been suggested [9].

Presently, 5 different Gzms have been described in humans: GzmA, GzmB, GzmH, GzmK, and GzmM. GzmA and GzmB have been studied more extensively than the other Gzms, which are therefore often referred to as orphan Gzms [2,10]. Despite their high sequence homology (approximately 40%), Gzms differ in their primary substrate specificity [1], thus providing CLs with various and redundant strategies to combat the wide range of viral infections. GzmA and GzmK cleave substrates after Arg or Lys (trypsin-like), GzmB after Asp or Glu (chymotrypsin-like), GzmH after Tyr or Phe (chymotrypsin-like), and GzmM after Leu or Met (elastase-like).

**Fig 1:**
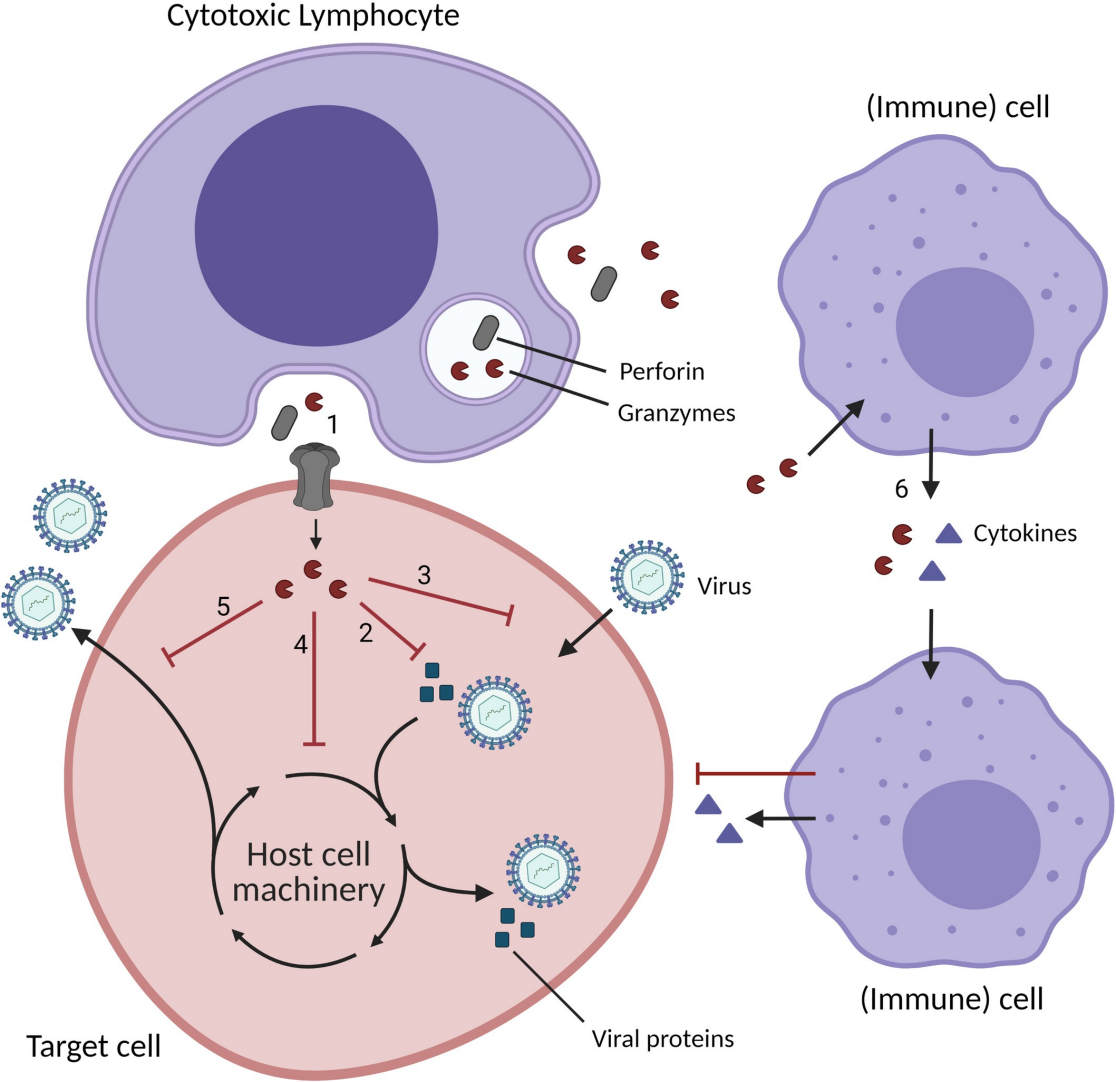
Schematic overview of the noncytotoxic functions of Gzms in viral infection. CLs secrete perforin and Gzms into the immunological synapse. Perforin pores mediate Gzm entry in the target cell (1). Gzms cleave and (in)activate viral proteins (2) and host cell proteins inside the cytoplasm and the nucleus necessary for viral entry (3), replication, protein synthesis, virion assembly (4), and viral release (5). CLs and also other cell types (e.g., macrophages) can directly secrete Gzms in the microenvironment. These extracellular Gzms can induce pro-inflammatory cytokine release by multiple cell types, such as monocytes, macrophages, DCs, mast cells, fibroblasts, and epithelial cells, further supporting the antiviral immune response (6). Created with BioRender.com. CL, cytotoxic lymphocyte; DC, dendritic cell; Gzm, granzyme.

Currently, cytotoxicity is considered to be the main antiviral function of all Gzms and especially of GzmB. Studies have shown that all human Gzms are capable of inducing target cell death; however, the cytotoxic potential of GzmA and the orphan Gzms has been highly debated [11–13]. Even highly cytotoxic GzmB can be essential to control viral infection without inducing apoptosis in infected cells [14]. How virus-infected cells escape apoptosis is still largely unknown but is likely the result of virus-mediated up-regulation or de novo expression of (viral) proteins that inhibit intracellular apoptosis pathways. [13,15]. Emerging evidence has shown that Gzm-mediated proteolysis of viral/host cell proteins can inhibit viral replication independently of cell death pathways (Table 1), a topic that has not been reviewed in the past decade. With this strategy, CLs can prohibit viral infection or suppress reactivation in latently infected cells [14,16]. Furthermore, Gzms can induce pro-inflammatory cytokine release supporting the antiviral immune response (Fig 1). In this review, an overview of the currently known noncytotoxic roles and potential therapeutic applications of Gzms in viral infections is provided.

**Table 1:** Validated Gzm substrates affecting viral replication upon cleavage.

Validated Gzm substrate	Gzm subtype	Noncytotoxic function during viral infection
*Host proteins*		
hnRNP A1	A [17]	Splicing inhibition [17], viral transcription and translation [18]
hnRNP A2/B1	A [17]	Transcription regulation [19], alternative splicing [20], viral RNA replication [21]
hnRNP C1/C2	A, B [17]	Viral RNA replication [22,23]
hnRNP-K	All human Gzms [24]	Transcription regulation [25–28], IE product expression [29], splicing inhibition, virion release [30]
hnRNP-U	A, B [17]	Promote immunity [31]
La	B, H [32]	Viral translation [33,34]
eIF4G3	B [35]	Viral translation [35]
RNA polymerase II	B [36]	Transcription, influenza virus replication [37]
B23/Nucleophosmin/numatrin	B [38], M [39]	Nuclear import, viral transcription, and virion assembly [40,41]
Topoisomerase I	B [36]	Reverse transcription [42]
Topoisomerase IIalpha	M [43]	Reverse transcription [44]
SET (SET-complex)	A [45], K [46]	Prevent autointegration [47], early gene transcription [48]
APE1 (SET-complex)	A [49], K [50]	Prevent autointegration [47]
HMG2 (SET-complex)	A [51], K [46]	Prevent autointegration [47]
Ku70	A [52], B [36]	Retroviral DNA integration [53]
DNA-PK	B [36,54]	Retroviral DNA integration [53]
PARP-1	A, B [55]	Viral replication [56,57]
Histone H1	A [58]	Stimulate/inhibit viral replication [59,60]
Lamin B	A, B [61]	Viral replication [62]
Importin *α*1	K [63]	Nuclear import [64]
Importin *β*	K [63]	Nuclear import [64]
α-tubulin	B [65,66], M [67]	Intracellular transport of viruses [68]
β-tubulin	K [69]	Intracellular transport of viruses [68]
Filamin A	B [70]	Arrangement of actin cytoskeleton [71]
*Viral*		
Adenovirus DBP	B, H [72]	DNA replication, transcription, mRNA stability, virus assembly [73]
GzmB-inhibitor 100K assembly protein	B, H [72]	Capsid assembly, GzmB inhibition [74,75]
pp71	M [76]	Viral replication, latency [76,77]
Hbx protein	H [78]	Viral replication [78,79]
HCMV IE1/2 protein	All 5 human Gzms [16]	Viral replication, latency [80–82]
HSV-1 ICP4	B [14] [83]	Early and late gene expression [84]
HSV-1 ICP27	B [83]	Late gene expression [85]
VZV ORF4	B [83]	Early and late gene expression [86], latency [87]
VZV ORF62	B [83]	Early and late gene expression [86]

DBP, DNA-binding protein; Gzm, granzyme; Hbx, hepatitis B virus x; HCMV, human cytomegalovirus; hnRNP, heterogeneous nuclear ribonucleoprotein; HSV-1, herpes simplex virus type I; IE, immediate early; ORF, open reading frame; PARP-1, poly(adenosine 5′-diphosphate [ADP]-ribose) polymerase-1; pp71, phosphoprotein 71; VZV, varicella zoster virus.

## Granzymes target viral proteins crucial for replication

Andrade and colleagues provided the first evidence of GzmH-mediated antiviral activity by cleavage of 2 adenoviral proteins: adenoviral DNA-binding protein (DBP) and GzmB-inhibitor 100K assembly protein [72]. DBP is abundantly present in adenovirus-infected cells and is essential for viral DNA replication. Furthermore, it has been associated with virion assembly and early and late viral gene expression [73,88]. Both GzmH and GzmB are able to inactivate DBP; however, GzmB cleaves much later during cytotoxic-mediated cell death than GzmH. Cells infected with mutant adenovirus encoding GzmH-resistant DBP initially demonstrated decreased DBP cleavage and higher viral loads post-killing compared to wild type. Later on, DBP was degraded by GzmB and viral loads subsequently decreased [72]. This could be explained by progressive inactivation of GzmB inhibitors, such as adenovirus 100K assembly protein [89]. This adenoviral protein is involved in multiple essential viral processes, such as viral assembly, inhibition of cellular translation, and activation of late viral translation [74,75]. Inactivation of 100K assembly protein by GzmH allows for the gradual recovery of GzmB activity [72], demonstrating the synergistic and redundant roles of Gzms to combat adenovirus infection.

GzmH was also proposed to play an important part in hepatitis B virus (HBV) replication and clearance without the induction of apoptosis. Once activated, GzmH disrupted HBV DNA replication in infected cells, while viral RNA synthesis remained unaffected. Furthermore, GzmH inhibitors abolished viral clearance, which is in concordance with a very low GzmH expression observed in chronic HBV carriers [78]. The effect of GzmH was suggested to be caused by cleavage of the viral HBx protein, a multifunctional regulator essential for viral replication [79]. GzmH colocalizes with HBx protein intracellularly and cleaves it at a highly conserved site. HBx-deficient HBV caused infections with low viral replication rates, which were resistant to lymphokine activated killer (LAK) cell- or GzmH-mediated viral clearance [78]. These results are in line with the hypothesis that GzmH-mediated proteolysis of HBx protein plays a vital part in controlling HBV infection.

Human cytomegalovirus (HCMV) is the most frequent viral cause of congenital defects and can provoke serious illness in immunocompromised patients. It can maintain a lifelong latent infection controlled by CLs secreting IFN-γ and Gzms. It has recently been shown that the killing capacity of HCMV-specific CTLs is low [90], suggesting that noncytotoxic processes play a rather important role for CL-mediated control of HCMV infection [16]. Recent studies have demonstrated that Gzms can influence HCMV replication independently of IFN-γ or cell death by directly and indirectly targeting the HCMV major immediate-early (MIE) gene products IE1 and IE2. These viral nuclear phoshoproteins play essential roles in initiating viral replication: IE2-deficient HCMV is noninfectious, and IE gene regulation has been associated with latency [80–82]. All human Gzms can directly inactivate IE1 and/or IE2 through cleavage at multiple specific sites [16]. Furthermore, GzmM can indirectly regulate IE expression by cleaving HCMV tegument phosphoprotein 71 (pp71).

During HCMV infection, the host cell protein Daxx silences the MIE promoter (MIEP), thus generating an intrinsic immune defense. However, HCMV pp71 translocates to the nucleus to degrade Daxx, thereby alleviating MIEP suppression. GzmM-mediated cleavage of pp71 blocks its ability to fight MIEP inhibition [76]. This is in line with the observation that GzmM-deficient mice are more susceptible to MCMV infection with higher viral burden [91]. Additionally, a relation between pp71 and latency has been suggested [13]. HCMV causes a latent infection in incompletely differentiated cells unable to accumulate pp71 in the nucleus, which is essential for the inactivation of Daxx. Reactivation occurs upon further differentiation toward a state with nuclear pp71 accumulation [13,77]. The redundancy with which human Gzms inhibit HCMV IE expression and function suggests that they employ important roles in controlling the HCMV infection.

Similar to HCMV, the alphaherpesviruses, such as herpes simplex virus type I (HSV-1) and varicella zoster virus (VZV), are known for their long periods of latent infection. During latent HSV-1 infection, CTLs surround infected neuronal cells and release GzmB without inducing neuronal damage [92]. GzmB- or perforin-deficient mice showed higher viral loads 14 days postinfection, indicating unstable latency, which returned to wild-type levels after, respectively, 20 or 34 to 36 days postinfection. This proposes a noncytotoxic role for GzmB in regulating HSV-1 infection. GzmB can directly cleave ICP4 [14], an HSV-1 immediate early (IE) protein required for early and late viral gene expression [84]. Also ICP27, an HSV-1 IE protein essential for late viral gene expression [85], can be cleaved by GzmB [83]. However, the biological relevance of the cleavage of ICP4 and ICP27 still needs further investigation. VZV is genetically similar to HSV-1 [93], but GzmB-expressing CTLs are in close proximity to VZV-infected cells only during active [94] but not latent infection [92]. GzmB is capable of cleaving the following VZV IE proteins: open reading frame (ORF) 4 and ORF62 (analogs of HSV-1 ICP27 and ICP4, respectively) [83]. ORF4 and ORF62 act in concert to transactivate early and late VZV genes, thereby playing crucial parts in viral replication [86]. In addition, ORF4 is essential to establish latent infection [87] and is capable of inhibiting NK cell–mediated killing, independent of its cleavage by GzmB [83]. Thus, GzmM is suggested to contribute to controlling alphaherpesvirus infection, although evidence under physiological conditions is still required.

## Granzymes target host proteins to influence/halt viral replication

The antiviral defense of Gzms is largely directed at host cell proteins hijacked by viruses, as viruses rely on the host cell’s machinery to replicate. Proteomic studies revealed large amounts of candidate Gzm substrates, although evidence for their inactivation by Gzms under physiological conditions is present only for a minority. In the past, research was mostly focused on the cytotoxic effects of their inactivation. However, many validated Gzm substrates play important roles in viral replication, suggesting that their inactivation could also have noncytotoxic antiviral effects. In the following section, we provide an overview of all host cell proteins that represent validated Gzm substrates with a potential to influence viral replication after cleavage (Table 1).

### Multifunctional host proteins involved in RNA metabolism

The majority of Gzm substrates are RNA/DNA binding proteins that are predominantly nuclear or can move toward the nucleus [17] and require nucleic acid binding for efficient cleavage [95]. This fits with the fact that Gzms can accumulate in the nucleus of target cells [96,97]. These substrates are often implicated in RNA metabolism, as underlined by the lack of nuclear export of newly transcribed RNA and disruption of pre-mRNA splicing in GzmA-treated cells [17]. This can serve as a vital antiviral weapon, as the viral life cycle of both DNA and RNA viruses rely heavily on this machinery. Below, we will discuss the multifunctional proteins, which have been most extensively studied in this regard.

Heterogeneous nuclear ribonucleoproteins (hnRNPs) are a large family of RNA-binding proteins (RBPs) involved in almost all steps of mRNA maturation [98] of which 6 isoforms are validated Gzm substrates [17,24]. Notably, all human Gzms can directly cleave the functional DNA/RNA binding domain of hnRNP K, and all, but GzmA, do so in living cells [24]. hnRNP K is a multifunctional protein involved in tumorogenesis whose normal cellular functions can be altered by interaction with DNA/RNA, IE, and core proteins of diverse viruses [99]. Inactivation of hnRNP K adds to the aforementioned redundant targetting of HCMV IE machinery by Gzms to reduce viral replication. For instance, hnRNP K–deficient fibroblasts infected with HCMV showed decreased levels of pan-Gzm substrate IE2 protein and subsequently lower viral loads compared to wild type. This is most likely caused by the crucial role of hnRNP K binding to IE mRNA for translation. Interestingly, hnRNP K–RNA binding is also required for the degradation of hnRNP K by Gzms, indicating a possible strategy to restrict the inhibition of replication to infected cells [29]. Additionally, hnRNP K binds to HBV DNA and the 5′ UTR of enterovirus 71 RNA and alters viral replication, as demonstrated by decreased viral loads upon lower hnRNP K expression [100,101]. During human immunodeficiency virus (HIV) infection, hnRNP K enhances viral transcription by forming a complex, including the Nef protein, to stimulate the viral transactivator Tat [25]. Furthermore, hnRNP K forms a complex with the GzmB substrate HSV-1 ICP27 (IE63), p32, and casein kinase 2 (ck2), which is suggested to inhibit splicing. This could contribute to host cell shutoff and redirection of nuclear transport to mostly intronless viral transcripts [102,103]. While hnRNP K knockdown did not affect HSV-1 DNA replication or transcript maturation, it significantly reduced virion release needed for viral propagation [30]. Human herpes virus 6 (HHV-6) IE2 protein also interacts with hnRNP K and ck2 [104], suggesting a role in HHV-6 viral life cycle. Additionally, the abundant African swine fever virus (ASFV) IE protein p30 causes nuclear redistribution of hnRNP K upon complexation and decreases cellular transcription [28]. Interaction of hnRNP K with the core proteins of hepatitic C virus (HCV) and dengue virus (DENV) has been suggested to diminish its inhibitory effects on transcription regulation, thereby disrupting its normal cellular functions to stimulate viral pathogenesis [26,27].

Other hnRNP isoforms are not as excessively targetted by different human Gzms. hnRNP A1, A2/B1, C1/C2, and U are all validated direct GzmA substrates, while GzmB cleaves hnRNP C1/C2 and U directly and hnRNP A1 and A2/B1 only through caspase activation [17]. hnRNP A1 is one of the most abundantly expressed multifunctional hnRNPs [105]. Overexpressed GzmA-resistant hnRNP A1 inhibits cell death and rescues splicing in cells treated with GzmA [17], suggesting an important role for hnRNP A1 in GzmA-induced cell death and splicing inhibition. Besides, hnRNP A1 influences viral replication by interacting with the nucleocapsid proteins of coronaviruses (SARS coronavirus, mouse hepatitis virus (MHV), and porcine epidemic diarrhea virus (PEDV)) [18]. It also binds to the 5′ UTR of the alphavirus prototype Sindbis virus, resulting into marked decreased viral RNA synthesis upon hnRNP A1 knockdown [106]. The effects of Gzms on the functions of hnRNPs A2/B1 and C1/C2 remain to be validated but are also potentially antiviral as these hnRNPs are critical for the life cycle of numerous viruses, particularly through interaction with viral proteins [19,21–23]. For example, HCMV IE2 protein interacts with hnRNP A2/B1 and stimulates its expression leading to cell proliferation and inhibition of apoptosis [20]. On the other hand, Gzm-mediated cleavage of hnRNP U is unlikely to have antiviral effects, as hnRNP U was suggested to promote immunity by activating antiviral enhancers [31].

La protein is also a multifunctional protein involved in RNA metabolism, as well as a major autoantigen associated with systematic autoimmune disease [107]. Moreover, it is required for the initiation of internal ribosome entry site (IRES)-mediated translation of viral gene products in cells infected with various viruses, including poliovirus, HIV1, encephalomyocarditis virus (EMCV), HCV, and coxsackievirus B3 [33,34]. La protein is cleaved by GzmB, GzmH, and caspases during CL killing. GzmH-induced truncated La 1–364 loses its nuclear localisation and has a dominant-negative effect on HCV-IRES mediated translation. Interestingly, phosphorylation of La at serine 366 inactivates its transcriptional function and makes it resistant to GzmH cleaving due to steric hindrance [32,108].

Eukaryotic initiation factor 4 gamma 3 (eIF4G3) is another host protein that is involved in viral translation and can be cleaved by GzmB. GzmB treatment of vaccinia virus (VV)-infected Jurkat cells decreased VV particle synthesis and eIF4G3 levels independently of caspase activity. VV particle production could partially be restored by expression of GzmB-resistant eIF4G3, but not wild-type eIF4G3, marking the importance of GzmB-mediated cleavage of eIF4G3 in halting viral protein synthesis [35].

Less is known about cleavage of RNA-synthesizing enzymes by Gzms. Two large subunits of RNA polymerase II can be cleaved by GzmB in intact cells [36], of which the biological relevance remains to be investigated. RNA polymerase II inactivation could inhibit transcription of DNA viruses and affect influenza viral replication by disrupting its interaction with viral RNA-dependent RNA polymerase required for the initiation of viral replication [37].

### Nucleolar host proteins

The nucleolus undergoes morphological and molecular modifications during infection with various viruses, which is important for numerous steps in the viral life cycle [109]. Nucleolar Gzm substrates nucleolin, nucleophosmin (B23), and fibrillarin are delocalized during viral infection. Nucleolin relocates most drastically, while being crucial for the regulation of nucleolar integrity and viral replication [36,109,110]. However, evidence of Gzm cleavage under physiological circumstances has only been found for B23, which is directly inactivated by GzmB and GzmM during Gzm-mediated killing of tumor cells [38,39]. The delocalization of B23 during adenovirus infection is facilitated by adenoviral protein V, and the knockdown of B23 reduces viral replication [41]. To add on, B23 is up-regulated during viral infection and stimulates the replication of viruses such as HIV, HBV, HCV, HDV, and HPV by influencing nuclear import, viral genome transcription, and virion assembly [40]. Gzm cleavage of B23 could therefore have a range of noncytotoxic repressing effects on viral infections.

### DNA repair machinery and retroviral DNA incorporation

Multiple host cell proteins important for DNA repair have been associated with Gzm-mediated apoptosis (topoisomerase IIalpha, SET complex, Ku70, DNA-PK, and PARP-1) [11,43]. Moreover, Gzm-mediated inactivation could also induce noncytotoxic effects as the DNA repair machinery is relevant for retroviral DNA incorporation and synthesis. For instance, reverse transcription of retroviral HIV-1 RNA into double-stranded DNA requires the presence of GzmB-substrate topoisomerase I [36,42] and GzmM-substrate topoisomerase II isoforms with the latter promoting RNA–DNA hybrids [43,44].

The viral DNA binds to the preintegration complex (PIC). For the transcribed retroviral DNA to be integrated in chromosomes, 3′ processing must occur to create reactive CA_OH_-3′ ends. However, CA_OH_-3′ ends are prone to attack sites within the viral DNA itself instead of the host DNA, resulting into suicidal autointegration [47]. The SET complex is a DNA repair complex containing 3 GzmA and GzmK substrates (SET, APE1, and HMGB2) that moves toward the nucleus during oxidative stress [11,69]. It inhibits autointegration by binding to the PIC. Consequently, knockdown of SET components leads to reduced HIV-1 chromosomal integration and viral replication [47]. Furthermore, the SET complex is involved in early gene transcription of adenovirus and DNA replication of adeno-associated virus [48,111].

The last step of retroviral DNA integration comprises joining of the 5′ end of viral DNA and the chromosome. HIV-1 integrase was suggested to interact with Ku70, thereby recruiting the DNA-PK complex to the integration site and subsequently stimulating the gap repair through nonhomologous end joining (NHEJ) [53]. Ku70 and DNA-PK are DNA repair proteins that can be cleaved during CL killing by GzmA and GzmB and exclusively GzmB, respectively [36,52,54]. Knockdown of Ku70 reduced HIV-1 postintegrational repair and viral replication [53,112], but the necessity of DNA-PK for HIV-1 integration has been debated [113–115].

Poly(adenosine 5′-diphosphate [ADP]-ribose) polymerase-1 (PARP-1), a single- and double-stranded DNA damage sensor, can be cleaved by GzmB in the nuclear localization signal and was proven to be inactivated by GzmA during CL attack [55]. It may compete with Ku70 to bind to double-stranded breaks for an alternative NHEJ pathway [116]; however, its importance in retroviral integration is controversial [115,117]. Moreover, PARP-1 showed opposing effects on viral replication: Its inhibition resulted in decreased HIV and John Cunningham (JC) virus DNA replication [56,57], while its activity has also been reported to repress retroviral transcription and lytic replication of oncogenic gammaherpesviruses [118,119]. To conclude, Gzms could have significant effects on retroviral integration and viral replication through targetting the DNA repair machinery; however, further research remains necessary.

### Host DNA replication machinery

Also, proteins involved in host DNA replication are degraded by Gzms. For instance, GzmA can disrupt the nuclear envelope through cleavage of lamins (A/C, B) and open up chromatins through histone H1 cleavage and removal of the tails of core histones [58,61]. This is paramount for Gzm-mediated cell death [15], however, these substrates have also been implicated in viral replication. For example, lamin B1 knockout mouse embryonic fibroblasts showed dramatically lower viral loads upon HSV infection compared to wild type [62]. In contrast, histone H1 has been suggested to stimulate or inhibit replication of different viruses [59,60], and lamin A/C was reported to be a key regulator in Th1 differentation upon VV and *Leishmania* major infection [120]. Therefore, the effect of Gzm cleavage of aforementioned substrates on viral replication is debatable and requires further research.

### Host proteins and intracellular viral transport

Finally, the intracellular transport of viral proteins is struck by Gzms through host protein targeting. Unlike many other RNA viruses, influenza A virus replicates inside the nucleus. Nuclear import of nucleoprotein (NP) and viral RNA polymerase subunits (PB1, PB2, and PA) is accomplished through interaction with host importin α/β dimers or a transport receptor related to importin β (β-importin Ran binding protein 5) [64]. GzmK cleaves the interaction domains of both importin α1 (Impα1) and importin β (Impβ) during CL killing, which disrupts their dimerization. Treatment of prepermeabilized Hela cells with recombinant Myc-NP and GzmK-truncated Impα1 (tImpα1) and Impβ (tImpβ) showed that nuclear import of NP was disrupted. Plus, tImpα1 and tImpβ could not rescue influenza polymerase activity in T293 cells with double knockdown of Impα1 and Impβ. Additionally, LAK cell–mediated clearance of influenza virus could be alleviated by GzmK inhibition, showing the importance of GzmK in reducing viral replication [63]. Moreover, the transport of viral particles to replication sites and their release demands an intact microtubule network comprised of polymerized α/β-tubulin heterodimers [68]. GzmB and GzmM can degrade α-tubulin, and GzmK cleaves β-tubulin during CL-mediated killing, leading to enhanced polymerization rates and aberrant microtubule networks [65–67,69]. Thereby, Gzms might delay viral propagation. Finally, GzmB was suggested to affect the arrangement of membrane proteins anchored in the cytoskeleton, which has been related to viral infection, by cleaving the actin filament cross-linking protein filamin A [70,71].

## Granzymes induce pro-inflammatory cytokine release

Besides specifically targeting viral/host proteins in infected cells, Gzms can also fight viral infections in a noncytotoxic manner through stimulation of (immune) cells. Recent research shows that all human Gzms, besides GzmH, can induce pro-inflammatory cytokine release, thus generating an antiviral immune response. Their role in inflammation is in line with the elevated levels of extracellular Gzms found in viral infections and other inflammatory diseases [6]. Moreover, perforin-deficient mice and patients suffering from mutations in the perforin gene, i.e., familial hemophagocytic lymphohistiocytosis, show enhanced cytokine response [121]. The following section portrays the relation between Gzms and the inflammatory in vitro and in vivo effects regarding viral infections for human GzmA, GzmB, GzmK, and GzmM.

### GzmA

Circulating plasma levels of GzmA are significantly elevated in patients infected with viruses such as Epstein–Barr virus (EBV), HIV-1, and chikungunya virus (CHIKV) [122,123]. This was also witnessed in mice infected with CHIKV and mouse models of zika virus (ZIKV) and DENV infections. Mice treated with GzmA inhibitor Serpinb6b showed drastically less foot swelling upon CHIKV infection. To add on, reduced foot swelling and NK and T cell infiltration was seen in GzmA−/− mice upon CHIKV infection with unchanged viral loads [124]. This proposes GzmA inhibition as a therapeutic strategy in fighting CHIKV-induced arthritis [124]. Furthermore, injection of recombinant mGzmA can induce edema and neutrophil infiltration in mice. Although the exact mechanism of extracellular GzmA’s pro-inflammatory function in vivo remains unclear, protease-activated receptor (PAR)-1 and PAR-2 might be involved. Foot swelling induced by recombinant mGzmA or CHIKV in mice could be diminished by PAR-1 and PAR-2 antagonists or the PAR-1 antagonist, Vorapaxor, respectively [123].

Recombinant hGzmA can directly stimulate pro-inflammatory cytokine release by several cell types in vitro. Extracellular GzmA induces the release of IL-8 by epithelial cells, IL-6 and IL-8 by fibroblasts, and IL-1β, tumor necrosis factor alpha (TNF-α), IL-6, and IL-8 by primary human monocytes [6,8]. These reactions are dependent on the catalytic activity of GzmA and enhanced when GzmA is delivered intracellularly. This indicates that GzmA’s proteolytic activity stimulates signaling and that the GzmA substrates responsible for the induction of these cytokines are most likely localized inside the cell [6]. Active, but not inactive, mGzmA can also stimulate IL-1β release by lipopolysaccharide (LPS)-preactivated primary mouse macrophages [8]. Although mGzmA can cleave and activate pro-IL-β in vitro [125], another study failed to reproduce these results with hGzmA [8]. IL-β maturation was suggested not to be caused by GzmA directly, but through involvement of the inflammasome, as caspase-1 inhibitors could diminish GzmA-mediated cytokine release by monocytes [8]. Furthermore, hGzmA potentiates cytokine release by primary human monocytes induced by a Toll-like receptor (TLR)-2 agonist or the bacterial TLR-4 agonist LPS, independently of catalytic activity. In contrast to earlier studies, GzmA alone failed to generate cytokine secretion by monocytes [126]. Whether viral TLR-2 or TLR-4 agonists, such as viral glycoproteins, also show this synergy with GzmA remains to be investigated.

Finally, extracellular GzmA can form the bridge between the innate immune system and the adaptive immune system by enhancing DC function. Recombinant GzmA stimulates phenotypic maturation and type I IFN release by plasmacytoid DCs (pDCs) and conventional DCs (cDCs) via the TLR9-MyD88 pathway. Large T cell responses were seen in mice vaccinated with antigen and GzmA, while pDC-depleted or IFN-α/βR-KO mice had a reduced T cell response after vaccination. This supports the importance of IFN-α producing pDCs in T cell activation and presents GzmA as a possible vaccine adjuvant [127].

### GzmB

Less is known about the inflammatory effects of GzmB, which is predominantly recognized for its cytotoxicity. GzmB levels in EBV and HIV-1 patients’ plasma and rheumatoid arthritis patients’ synovial fluid are elevated, although consistently lower than GzmA levels [122]. In vitro studies on GzmB’s cytokine inducing capacities are limited. hGzmB is unable to induce IL-6 and IL-8 production by Hela cells or HUVECs directly and can only potentiate LPS-mediated TNF-α release by human monocytes. On the other hand, it can convert pro-IL-18 into active IL-18 in vitro and ex vivo, similarly to caspase-1 but with slower kinetics. Additionally, it can cleave the 31 kDa precursor of IL-1α, thereby increasing its biological activity, as demonstrated in vitro and in a mouse model [6]. To conclude, GzmB’s pro-inflammatory role is largely based on cytokine processing instead of initiation.

### GzmK

GzmK shows comparable inflammatory responses to GzmA. Similarly, extracellular hGzmK induces IL-6 and IL-8 release by fibroblasts, while the release of chemokine MCP-1 has also been reported [7]. This is dependent on GzmK’s catalytic activity as well as its intracellular delivery, and it involves cleavage of PAR1. Furthermore, like GzmA, active recombinant mGzmK stimulates IL-1β production by LPS-preactivated macrophages [6]. IL-1 signaling is crucial to fight lymphocytic choriomeningitis virus (LCMV) infection, as shown by the significant decrease of LCMV elimination in mice treated with IL-1 receptor antagonist, anakinra, with unaffected T cell inactivation [128]. This could explain the redundant roles of different Gzms in IL-1 maturation. However, GzmA-GzmB–deficient mice and their CLs, expressing mostly GzmK with little proapoptotic activity, as well as GzmK-deficient mice could all control LCMV infection [128,129]. This suggests that the noncytotoxic roles of GzmK can contribute to the immune response against LCMV, but are not essential. Additionally, hGzmK works synergistically with TLR-4 agonist LPS to generate TNF-α, IL-6, and IL-8 release by monocytes, independently of proteolytic activity as observed with GzmA [130]. In vivo results showed that GzmK−/− mice also exhibit reduced foot swelling following CHIKV infection, although less drastically than GzmA−/− mice [124].

### GzmM

Research into the inflammatory functions of GzmM has mostly been focused on bacterial infections. However, a recent study showed that GzmM levels were elevated in the synovial fluid of RA patients, which correlated with high local cytokine levels. Furthermore, hGzmM could trigger IFN-λ1 (IL29) release by human fibroblasts, completely dependent on its catalytic activity [131]. This could bestow potential antiviral effects to GzmM as type III IFNs, such as IFN-λ1, play crucial roles in restricting replication of various viruses in vitro and in vivo [132]. Furthermore, GzmM colocalizes with MIP-1α in the cytotoxic vesicles of human NK cells [6]. The chemoattractant protein MIP-1α stimulates inflammation by recruiting monocytes, NK cells, neutrophils, and antigen-specific T and B cells. MIP-1α–deficient mice showed delayed clearance of some viral infections, including influenza virus, but mostly decreased inflammation-mediated damage upon viral infection [133]. NK cells and macrophages isolated from the livers of GzmM-deficient and LPS-challenged mice showed less MIP-1α secretion than wild type. This suggests a role for GzmM to increase local MIP-1α secretion upon bacterial infection [6]. It remains to be investigated if viral infections lead to similar results and which mechanism is involved.

## Conclusions and potential therapeutic consequences

The fight against viruses is an enormous topical challenge with worldwide impact. Viral infections can spread rapidly and have devastating consequences, especially in immunocompromised patients, while effective treatment against numerous viruses is still lacking. Understanding how the healthy immune system controls viral infections can aid in the development of future therapies. Emerging evidence shows that killer cells use Gzms in viral infections not only to kill target cells, but also to employ multiple noncytotoxic functions. These noncytotoxic functions are likely relevant when viruses employ inhibitors of (Gzm-induced) apoptosis and/or during the attack of immune privileged sites such as neurons. Cleavage of some of these Gzm substrates have been directly demonstrated to affect viral replication in vivo. For example, Gzm-mediated inactivation of the substrates adenovirus DBP and host cell proteins La protein and eIF4G3 is directly proven to decrease viral replication, translation, or viral particle synthesis [32,35,72]. Many other validated Gzm substrates play vital roles in viral replication, although more research is often required to confirm the noncytotoxic antiviral effects of Gzm-mediated inactivation in physiological settings. The growing list of these Gzm substrates marks potential therapeutic targets. However, attacking host cell proteins hijacked by viruses might impose problems as these proteins also play crucial roles in uninfected cells. This challenge illustrates the potential of targeted therapy. In the recent years, GzmB has been extensively studied as an immunotoxin in relation to targeted cancer therapy. Specific delivery of GzmB in cancer cells has been achieved, for instance, by genetic fusion with an antibody moiety or a derivative of natural ligands binding surface cell proteins or receptors. GzmB inhibition by serpin B9 (PI-9) and the binding of highly basic GzmB to the extracellular matrix are other obstacles in GzmB therapy. Promising solutions for these problems involve mutating the GzmB–PI-9 interaction site and basic surface amino acids, respectively [134,135]. It would be interesting to investigate whether similar tactics can be used in relation to viral infections.

Therapeutic strategies can also be based on the immunostimulatory effects of Gzms. For example, GzmA could be added to vaccines to increase their effectiveness by stimulating the innate immune system, as mentioned before [127]. On the other hand, Gzm inhibitors may be powerful remedies against the damage bestowed by inflammation seen in viral infections. As described afore, GzmA inhibition could repress CHIKV-mediated arthritis [124], and GzmM might also contribute to inflammation-mediated damage [6,133]. The paradoxical consequences of Gzms’ pro-inflammatory effects during viral infections emphasize the importance of extensive in vitro and in vivo studies on the matter.

To conclude, cytotoxic cells use Gzms to play important noncytotoxic roles in the fight against viral infections, by cleaving viral/host proteins essential for viral replication and generating an antiviral immune response. This information provides a better understanding of the natural antiviral defense and proposes future therapeutic strategies.
